# Aggression Profiles in the Spanish Child Population: Differences in Perfectionism, School Refusal and Affect

**DOI:** 10.3389/fnbeh.2018.00012

**Published:** 2018-01-30

**Authors:** María Vicent, Cándido J. Inglés, Ricardo Sanmartín, Carolina Gonzálvez, José Manuel García-Fernández

**Affiliations:** ^1^Department of Developmental Psychology and Didactics, Faculty of Education, University of Alicante, Alicante, Spain; ^2^Department of Clinical Psychology, Faculty of Social-Health Sciences, Miguel Hernández University of Elche, Elche, Spain

**Keywords:** aggressive behavior, profiles, childhood, socially prescribed perfectionism, self-oriented perfectionism, school refusal, positive affect, negative affect

## Abstract

The aim of this study was to identify the existence of combinations of aggression components (Anger, Hostility, Physical Aggression and Verbal Aggression) that result in different profiles of aggressive behavior in children, as well as to test the differences between these profiles in scores of perfectionism, school refusal and affect. It is interesting to analyze these variables given: (a) their clinical relevance due to their close relationship with the overall psychopathology; and (b) the need for further evidence regarding how they are associated with aggressive behavior. The sample consisted of 1202 Spanish primary education students between the ages of 8 and 12. Three aggressive behavior profiles for children were identified using Latent Class Analysis (LCA): *High Aggression* (*Z* scores between 0.69 and 0.7), *Moderate Aggression* (*Z* scores between −0.39 and −0.47) and *Low Aggression* (*Z* scores between −1.36 and −1.58). These profiles were found for 49.08%, 38.46% and 12.48% of the sample, respectively. *High Aggression* scored significantly higher than *Moderate Aggression* and *Low Aggression* on Socially Prescribed Perfectionism (SPP), Self-Oriented Perfectionism (SOP), the first three factors of school refusal (i.e., FI. Negative Affective, FII. Social Aversion and/or Evaluation, FIII. To Pursue Attention), and Negative Affect (NA). In addition, *Moderate Aggression* also reported significantly higher scores than *Low Aggression* for the three first factors of school refusal and NA. Conversely, *Low Aggression* had significantly higher mean scores than *High Aggression* and *Moderate Aggression* on Positive Affect (PA). Results demonstrate that *High Aggression* was the most maladaptive profile having a high risk of psychological vulnerability. Aggression prevention programs should be sure to include strategies to overcome psychological problems that characterize children manifesting high levels of aggressive behavior.

## Introduction

Aggression has been typically defined as a “behavior directed toward harming or injuring another living being who is motivated to avoid such treatment” (Blair, 2016, p. 4). Although the study of aggression was originally limited to its direct physical and verbal forms (Archer, 2009), it is currently considered to be a complex construct involving multiple components, forms and functions. This study is based on the conceptualization of aggression as proposed by Buss and Perry (1992) who consider aggressive behavior to be the combination of three components: emotional (Anger), cognitive (Hostility) and motor (Physical and Verbal Aggression). Anger is an emotion that involves feelings of variable intensity ranging from mild irritation to intense fury (Lubke et al., 2015). Hostility refers to a cognitive state consisting of attitudes and feelings of negative evaluation toward others, such as cynicism, mistrust and suspicion (Fabiansson and Denson, 2016). Lastly, the motor component of aggression implies any physical or verbal action that is carried out in order to injure others (Leary et al., 2006).

Far from being an exclusive manifestation of adolescence or adulthood, aggressive behavior may develop from a very early age (Hay, 2017) and is one of the most common causes for child therapy referrals (Shachar et al., 2016). Over recent years, research on aggressive behavior during childhood has become particularly relevant. This is due in part to the high prevalence of aggressive manifestations in the child population, such us bullying (Modecki et al., 2014), as well as the adverse consequences derived from each of these three components (cognitive, emotional and motor), including a tendency to develop physical and mental health problems, drug use, delinquency, etc. (e.g., Harachi et al., 2006; Kerr and Schneider, 2008; Hampson et al., 2010; Garaigordobil et al., 2013). Furthermore, aggressive behavior has been also identified as having a putative modulating role between genetic factors and the emergence of suicidal behavior in psychosis (Serafini et al., 2012).

Research on the stability of aggression tends to reveal more patterns of continuity than discontinuity (Piquero et al., 2012). Advances in statistical methods for data modeling have evidenced a nonlinear continuity of aggression, also revealing the existence of certain risk factors that explain variance in aggression that is above and beyond its continuity from childhood to adulthood (Petersen et al., 2015). This continuity is particularly strong over time in individuals manifesting early highly aggressive behavior (Piquero et al., 2012). Therefore, identifying groups of aggressive behavior is of particular importance during the school age, allowing for the examination of the psychological profile of children exhibiting high levels of aggression. Furthermore, individual and contextual differences between high and low aggression groups may provide clues as to both protective and predisposing variables that should be considered in intervention/prevention actions for highly aggressive individuals. More specifically, this study analyzed the role of perfectionism, school refusal and affect as possible mitigating or enabling factors of such tendencies.

The interest in studying these three constructs is mainly due to their close link with overall psychopathology. Thus, perfectionism has even been considered as a transdiagnostic process (Egan et al., 2011, 2012). Specifically, with respect to child perfectionism, it has been conceptualized according to two dimensions (Flett et al., 2016): Socially Prescribed Perfectionism (SPP), understood as the tendency to consider the environment as highly demanding of perfectionism; and Self-Oriented Perfectionism (SOP), which captures the tendency to be sharply self-critical and to impose excessive high performance goals on oneself.

Second, although there is a declining trend in overall school dropout rates (Freeman and Simonsen, 2015), high levels of this problematic prevail. Moreover, advances in the reduction of school dropout rates has not taken place equally in terms of cultural and socioeconomic aspects. This situation justifies the relevance of addressing cases of school refusal which are, in turn, associated with multiple internalizing and externalizing problems (e.g., Maynard et al., 2012; Ingul and Nordahl, 2013). School refusal refers to the “avoidance of a child attending school and/or persistent difficulty staying in the classroom throughout the school day” (Inglés et al., 2015, p. 37). According to the functional model of Kearney and Diliberto (2014), the school refusal behavior may be explained based upon four reasons or factors which are not mutually exclusive: FI. To Avoid Negative Affectivity (associated with younger students who refuse to attend school and have difficulties identifying the cause of their discomfort); FII. To Avoid Social Aversion and/or Evaluation (linked with those students who present social difficulties and suffer in assessment situations, such as exams or oral presentations); FIII. To Pursue Attention (related to those students who prefer staying at home or with their parents or loved ones instead of going to school); and FIV. To Pursue Tangible Reinforcement (characterized by truancy based on the desire to engage in leisure activities outside of the educational center, such us staying at home watching the TV, playing computer games or spending time with friends).

Third, Positive affect (PA) is a positive, energetic, emotional, affiliation and dominion dimension of an individual, whereas Negative Affect (NA) is characterized by mood states such as sadness, aversion, anger, contempt, disgust, guilt, fear and nervousness (Watson et al., 1988; Clark et al., 1994). Both dimensions have been widely used by research as indicators of adjustment and maladjustment, respectively (e.g., Schütz et al., 2013; Liu et al., 2014).

However, despite the importance of these three variables, previous studies examining the relationship between perfectionism, school refusal, affect and aggressive behavior are limited or non-existent. Regarding perfectionism (i.e., SPP and SOP), García-Fernández et al. (2017) were the first to analyze the association between SPP and aggressive behavior in accordance with the model of Buss and Perry, using a Spanish sample of students aged 8–11. Results revealed that all components of aggressive behavior were significant and positive predictors of high levels of SPP. Likewise, students with high levels of SPP scored significantly higher on all components of aggressive behavior than their peers with low levels of SPP. Also, regarding Spanish child population, Vicent et al. (2017) found that a cluster defined by high SPP and SOP obtained significantly higher levels of Anger, Hostility and Physical and Verbal Aggression than any other combination of SPP and SOP. Lastly, Stoeber et al. (2017) used bivariate and partial correlations (controlling for the effect of the other perfectionist facets) in three samples of English university students. Thus, while the results indicated a positive and significant relationship between SPP and all components of aggressive behavior, except for Verbal Aggression; SOP obtained positive and significant bivariate correlations with Hostility and Verbal Aggression, as well as negative and significant partial correlations with Physical Aggression.

On the other hand, no study to date has examined the relationship between the functional model of school refusal and the aggression model of Buss and Perry (1992). However, research has revealed how this type of aversion to the school may influence the development of the aggressive behavior. For instance, Wallinius et al. (2016), in a sample of Swedish prisoners, found that school absenteeism at early ages was one of the major predictors of antisocial behaviors during adulthood. Similarly, other studies with American clinical child and/or adolescent populations have agreed to positively link FIII. To Pursue Attention and FIV. To Pursue Tangible Reinforcement with externalizing behaviors (Kearney and Silverman, 1993; Higa et al., 2002; Kearney, 2002). Specifically, Kearney and Silverman (1993) found positive and significant correlations between FIII and FIV and externalizing behavioral problems. Nevertheless, in the study of Higa et al. (2002), these correlations were only significant for FIV. Similarly, Kearney (2002) also concluded that both externalizing and internalizing problems jointly prevailed in FIII, whereas only externalizing problems prevailed in FIV.

Lastly, of the two affective dimensions, NA is the most closely linked with the aggressive behavior (Donahue et al., 2014). Based on the model of Buss and Perry, several studies have examined how both affective dimensions have been linked with aggression. Specifically, Verona et al. (2002), in a sample of American undergraduates, found that participants with high negative emotion scored significantly higher on all components of aggressive behavior as compared to their peers with low negative emotion. Considering only the Anger dimension, Harmon-Jones (2003) found positive and significant correlations between Anger and NA, as well as non-significant correlations with PA, in a sample of American undergraduate students. In contrast, Hewig et al. (2004), using German undergraduates, observed that PA was significantly and negatively associated only with Hostility, whereas NA was not significantly linked with Anger, Hostility and Physical and Verbal Aggression. Similarly, Dufey and Fernández (2012), in a sample of Chilean undergraduates, found that PA was negatively and significantly linked with all of the components of aggressive behavior, whereas NA was linked in a positive sense. More recently, Shachar et al. (2016), in a sample of Israeli students (grades 3–6) with observed aggressive behavior, obtained positive and significant correlations between NA and Anger, Hostility and Physical Aggression, with no data provided regarding Verbal Aggression. In contrast, such correlations were negative and significant between PA and Hostility and Physical Aggression, whereas the relationship with Anger was not statistically significant.

This study has a two-fold goal. First, it aims to verify whether there are different profiles of students with aggressive behavior, considering the cognitive, emotional and motor components established by Buss and Perry (1992). On the other hand, some important questions about the relationship between aggressive behavior and perfectionism, school refusal and affect remain unanswered. Thus, no study to date has examined the link between SOP, Positive and NA and the three components of aggressive behavior during childhood. Regarding school refusal, there is no previous empirical evidence about how the four functions of school refusal are associated with aggressive behavior. So, in order to overcome this limitations, after the identification of the profiles of aggressive behavior, the significant differences between the profiles identified on perfectionism (i.e., SPP and SOP), the four factors of school refusal, and Positive and NA are determined.

Thus, it is expected that profiles with more levels of aggressive behavior shall obtain: (a) *Hypothesis 1*. Significantly higher levels in SPP and SOP, in accordance with those studies that suggest a positive relationship between both perfectionist dimensions and all or some of the components of the aggressive behavior (García-Fernández et al., 2017; Stoeber et al., 2017; Vicent et al., 2017); (b) *Hypothesis 2*. Significantly higher scores in FIII and FIV on school refusal, in line with previous research that has identified a positive relationship between these factors and externalizing problems (Kearney and Silverman, 1993; Higa et al., 2002; Kearney, 2002); and (c) *Hypothesis 3*. Significantly lower scores on PA and higher on NA, in accordance with previous works that observed a negative and significant association between PA and some components of the aggressive behavior (Hewig et al., 2004; Dufey and Fernández, 2012; Shachar et al., 2016), as well as positive in the case of NA (Verona et al., 2002; Harmon-Jones, 2003; Dufey and Fernández, 2012; Shachar et al., 2016).

## Materials and Methods

### Participants

The sample was recruited using multi-stage random cluster sampling, which means that all clusters were randomly selected in each stage, in the geographic areas: central, north, south, east and west of the Alicante province (Spain). Between one and three centers were randomly and proportionally selected from each geographical zone. Thus, a total of 16 schools were selected. From each of these schools, one group per academic grade was randomly selected from 3rd to 6th grade of primary education. Following this procedure, an initial sample of 1397 students was obtained, of which 195 were excluded because: (a) their parents and/or legal guardians did not give written contest to participate in the study (*N* = 74); (b) they did not have the minimum reading level required; (*N* = 68); or because; of (c) errors or omissions in the questionnaire completion (*N* = 53). Thus, the final sample consisted of 1202 Primary Education students aged 8–12 (*M*_age_ = 10.25, *SD* = 1.28). 48.6% of participants were males and 51.4% were females. Sample distribution across age was: 12.6%, 17.3%, 20.3%, 32.3% and 17.5%, respectively for students from 8 to 12 years. As for ethnic composition, 88.1% were Spanish, 5.9% South American, 4.7% Arab and 1.3% were of other origins.

### Instruments

#### The Aggression Questionnaire (AQ; Buss and Perry, 1992)

The Aggression Questionnaire (AQ) is a 29-item self-report measure with a 5-point Likert scale of four dimensions of aggressive behavior: Anger, Hostility, Physical Aggression and Verbal Aggression. Specifically, the Spanish version of the scale validated by Santisteban and Alvarado (2009), whose levels of reliability range from *α* = 0.65 (Anger) to 0.80 (Physical Aggression), was used. Acceptable internal consistency indices were obtained in this study: *α* = 0.71, 0.73, 0.80 and 0.75, respectively, for Anger, Hostility, Physical Aggression and Verbal Aggression.

#### The Child-Adolescent Perfectionism Scale (CAPS; Flett et al., 2016)

The Child-Adolescent Perfectionism Scale (CAPS) is a 22-item self-report measure with a 5-point Likert scale of SPP and SOP. It is the most widely employed measure to assess child perfectionism (García-Fernández et al., 2016). Specifically, the Spanish translation of the scale provided by Castro et al. (2004), whose levels of reliability were *α* = 0.82/92 (SPP) and *α* = 0.75/92 (SOP), was used. Acceptable internal consistency indices were obtained in this study: *α* = 0.74 for SPP and 0.76 for SOP.

#### The School Refusal Assessment Scale Revised for Children (SRAS-R; Kearney, 2002)

The School Refusal Assessment Scale Revised for Children (SRAS-R) is a 24-item self-report measure with a 7-point Likert scale of four functions of school refusal: FI. To Avoid Negative Affectivity, FII. To Avoid Social Aversion and/or Evaluation, FIII. To Pursue Attention, and FIV. To Pursue Tangible Reinforcement. In this study, the Spanish version developed by Gonzálvez et al. (2016), whose levels of reliability range from *α* = 0.70 (FI) to 0.87 (FIII), was employed. Acceptable internal consistency indices were obtained in this study: 0.72, 0.74, 0.76 and 0.71, respectively, for the four factors of the SRAS-R.

#### The Positive and Negative Affect Schedule for Children (10-Item PANAS-C; Ebesutani et al., 2012)

The 10-Item Positive and Negative Affect Schedule for Children (10-Item PANAS-C) is a self-report measure with a 5-point Likert scale of PA and NA. Internal consistency levels of 0.86 and 0.82 were obtained for PA and NA (Ebesutani et al., 2012). The 10-Item PANAS-C was translated to Spanish using a back-translation method, in accordance with the recommendations of Hambleton and Lee (2015). Acceptable internal consistency indices were obtained in this study: 0.78 and 0.82, respectively, for PA and NA.

### Procedure

A meeting was held with the head teachers of the selected educational centers in order to inform them of the aims of the study and to request their collaboration. All centers agreed to cooperate in the investigation. Subsequently, the written informed parental consent was requested from all participants. Since the study participants were minors, a letter describing the aims of the study was provided to parents and/or legal guardians of the students selected to participate. The letter included a section that parents and/or legal guardians were to sign and return to the school in case they give their consent to participate in the study. Only those minors who provided the parental consent participated in the study. The four tests were administrated in a single 60-min session, in which a researcher was present. During test administration, the researcher highlighted the strictly voluntary and anonymous character of the activity. Participants did not receive compensation for their contribution to this study.

This research was carried out in accordance with the recommendations of the ethical standards of the 1964 Helsinki Declaration and its subsequent amendments. The protocol was approved by the Ethics Committee of the University of Alicante (Spain) (UA-2017-09-05). Written parental informed consent was obtained from all parents or legal guardians of minors participating.

### Data Analysis

The profiles of child aggressive behavior were defined based on the different combinations of Anger, Hostility, Physical Aggression and Verbal Aggression. To determine the number of profiles, a Latent Class Analysis (LCA) was performed. LCA is a model-based technique that is currently considered to be the best method of identifying homogeneous classes of subjects given that it overcomes all of the problems related to K-means clustering (Schreiber, 2017). Data-driven calculations begin with one class. Individuals are then successively allocated to an ascending number of classes. The fit indices and the criteria taken into account when choosing the most adequate class solution were the lowest Bayesian Information Criteria (BIC) and Entropy values closer to one (Nyland et al., 2007; Schreiber, 2017; Smeets et al., 2017).

Once the aggressive behavior profiles were established, the inter-class differences in the obtained scores on perfectionism, school refusal and affective dimensions were analyzed using the analysis of variance (ANOVA). Moreover, gender was included as a covariate to analyze its moderator effect. The* Scheffé* method was used to analyze the *post hoc* tests, as well as the Cohen’s *d* index to calculate the effect size of the observed differences. Specifically, *d* levels between 0.20 and 0.49 indicate a small effect magnitude; between 0.50 and 0.79 indicate a moderate magnitude; and ≥0.80, a large one (Cohen, 1988).

## Results

### Aggressive Behavior Profiles

**Figure 1:**
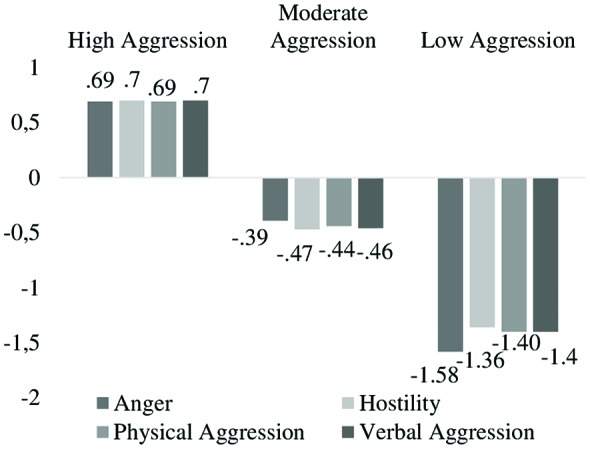
Graphic representation of the profiles of child aggressive behavior through latent class analysis (LCA).

The LCA allowed for the identification of three profiles characterized by different levels of aggressive behavior (see Figure 1). As shown in Table 1, this three-class model was the best-fitting, having the lowest BIC and the highest entropy. The first class, *High Aggression*, included 590 students (49.08%) having high levels in all components of aggressive behavior. The second class, *Moderate Aggression*, consisted of 462 participants (38.46%) characterized by moderate levels in all components of aggressive behavior, whereas the third class, *Low Aggression*, classified 150 students (12.48%) with low levels of aggressive behavior.

**Table 1:** Fit indices of the latent class analysis (LCA) values in bold show the best model fit.

Amount of classes	BIC	Entropy
2 classes	11875.73	0.84
**3 classes**	**10777.86**	**0.86**
4 classes	10836.91	0.83
5 classes	10890.47	0.79
6 classes	10896.93	0.76

### Inter-class Differences in Perfectionism, School Refusal and Affect

The results of the ANOVA indicate the existence of statistically significant differences between the three profiles of aggressive behavior for all of the variables considered in this study, with the exception of FIV of the SRAS-R (see Table 2). These differences were maintained when gender was included as a covariate, i.e., still a significant main effect of class.

**Table 2:** Mean scores, standard deviations and *post hoc* contrasts between mean perfectionism, school refusal and affect scores obtained by the three classes of aggressive behavior.

Variable	High aggression		Moderate aggression		Low aggression		Statistical significance		
	*M*	*SD*	*M*	*SD*	*M*	*SD*	*F*_(2,1199)_	*p*	*η*^2^
SPP	32.09	8.01	29.48	7.89	28.04	7.15	10.21	<0.001*	0.04
SOP	41.63	7.69	39.56	7.22	38.50	9.64	7.14	<0.001*	0.02
FI	7.52	5.67	4.36	4.15	1.72	2.09	46.37	<001*	0.11
FII	4.73	5.67	2.23	3.38	1.01	1.79	30.24	<001*	0.07
FIII	12.62	7.44	10.85	6.92	7.36	4.68	9.81	<001*	0.03
FIV	12.32	4.14	12.23	4.60	11.90	3.74	0.115	0.892*	0.01
PA	19.64	4.11	19.02	3.59	21.07	4.25	9.21	<001*	0.03
NA	9.73	4.34	7.55	3.50	5.44	0.704	27.99	<001*	0.09

Note: FI, Factor I. To Avoid Negative Affectivity, FII, Factor II. To Avoid Social Aversion and/or Evaluation, FIII, Factor III. To Pursue Attention, FIV, Factor IV. To Pursue Tangible Reinforcement, PA, Positive Affect; NA, Negative Affect. *Also when gender was considered as a covariate.

Specifically, *post hoc* comparisons revealed that *High Aggression* obtained significantly higher mean scores in SPP, SOP, FI, FII and FIII for school refusal and NA as compared to *Moderate* and *Low Aggression*. The comparisons between *Moderate Aggression* and *Low Aggression* were significant in the case of the three first factors of the SRAS-R and NA. Finally, *High Aggression* and *Moderate Aggression* scored significantly lower on PA than *Low Aggression*.

The magnitude of these differences (Cohen’s *d* index) ranged from 0.24 to 1.36. Differences between *High Aggression* and *Low Aggression* revealed the largest effect sizes (see Table 3).

**Table 3:** Cohen’s *d* index to *post hoc* contrasts between the mean scores obtained and the three classes in the factors of perfectionism, school refusal and affect.

	High aggression vs. Moderate aggression	High aggression vs. Low aggression	Moderate aggression vs. Low aggression
SPP	0.33	0.40	n.s.
SOP	0.28	0.36	n.s.
FI	0.64	1.36	0.81
FII	0.54	0.87	0.43
FIII	0.24	0.85	0.59
FIV	n.s.	n.s.	n.s.
PA	n.s.	0.34	0.52
NA	0.55	0.73	0.38

*Note: FI, Factor I. To Avoid Negative Affectivity, FII, Factor II. To Avoid Social Aversion and/or Evaluation, FIII, Factor III. To Pursue Attention, FIV, Factor IV. To Pursue Tangible Reinforcement, PA, Positive Affect; NA, Negative Affect; n.s., non-significant differences*.

## Discussion

Three profiles of child aggressive behavior (*High Aggression*, *Moderate Aggression* and *Low Aggression*) characterized, respectively, by high, moderate and low levels on all AQ dimensions (i.e., Anger, Hostility, Physical and Verbal Aggression) were identified. These results indicate that although such dimensions reflect different components of aggression, a close and positive relationship exists among them (McKay et al., 2016). Thus, the complexity of aggressive behavior is evident, extending beyond the motor component (i.e., Physical and Verbal Aggression) and it also implies the hostile beliefs and cognitions system of the subject as well as their wrathful emotional tendencies.

Regarding the results for each class, inter-class differences were found for all of the dimensions considered in this study, with the exception of the FIV on school refusal. *High Aggression* reported higher scores on all maladjustment dimensions, as well as the lowest levels in PA, proving to be the most maladaptive group. In contrast, *Low Aggression* obtained the best results in terms of psychological adjustment. As for the third profile, *Moderate Aggression* emerged as the second most maladaptive profile. These results question the premise that certain levels of aggression may become adaptive and demonstrate that children with a low aggressive behavior profile tend to be better adjusted psychologically. Thus, although certain authors have attributed some benefits to aggression for the resolution of certain social problems (Pellegrini, 2007), aggressive behavior should be considered both dangerous and time-consuming (Nelson and Trainor, 2007).

Similarly, differences in the mean scores in perfectionism, school refusal and affect between the three profiles identified in this study allow for the examination of the relationship between such variables and aggressive behavior.

First, with respect to both perfectionist dimensions, students characterized by high levels of aggression scored significantly higher on SPP and SOP than their peers with low and moderate levels of aggressive behavior. These results are in accordance with *Hypothesis 1* and with those studies that found evidence of a positive association between the perfectionist dimensions and the components of aggressive behavior (García-Fernández et al., 2017; Stoeber et al., 2017; Vicent et al., 2017). However, effect sizes of these differences have shown that aggressive behavior is more closely linked to SPP than to SOP. García-Fernández et al. (2017) explained the relationship between SPP and aggression based on the frustration-aggression model. According to these authors, children with high SPP manifest aggressive behaviors, either physical or verbal, for two reasons. On the one hand, as a consequence of the anger experienced toward others after the humiliation resulting from not being able to achieve the imposed expectations. On the other hand, it could be justified as an attempt to defend themselves from an environment that is considered to be highly harsh and critical, in other words, as a consequence of the hostility. Likewise, the frustration resulting from failing to reach the high self-imposed goals and the anger derived from a strong tendency to self-criticism would explain the link between SOP and aggressive behavior (Vicent et al., 2017).

Regarding the relationship between the aggression profiles and the explanatory factors of school refusal, the results partially support *Hypothesis 2*, since students characterized by high levels of aggression scored significantly higher on the first three factors of the SRAS-R as compared to their peers with low levels of aggressive behavior. The large differences between *High* and *Low Aggression* in FIII are expected, given that this third factor of school refusal has been associated with both internalizing and externalizing problems (Kearney, 2002). However, the effect sizes revealed that the largest differences between the *High* and *Low Aggression* profiles were associated with the first two factors of the SRAS-R, which present comorbidity with generalized anxiety, social anxiety and depression (Kearney and Albano, 2004; Kearney et al., 2005). These findings contrast with previous research that has associated externalizing problems with FIII and FIV, mainly, with subjects who base their school refusal on FIV due to its relationship with school absenteeism. FIV is more frequent in adolescents than in children. It is not based on anxiety and is developed without parental consent (Yahaya et al., 2010; Kearney, 2016). However, a number of emotional reactions such as excessive anxiety, crying, stress, or excessive somatic complaints may also arise, especially in younger students who base their school refusal on FI and FII (Kearney and Bensaheb, 2006), which could be accompanied by an aggressive behavior response. In fact, in a review performed by Grant et al. (2004) it was noted that in 53 of the 60 studies reviewed, general stress levels predicted high levels of aggressive behavior.

Despite the fact that scientific literature has revealed a positive and significant correlation between the FIII and FIV on school refusal and the presence of externalizing behaviors (Kearney and Silverman, 1993; Higa et al., 2002), none of the previous studies was carried out in a Spanish community sample of participants in the late childhood period (8–12 years of age) and applying an instrument of aggressive behavior based on components at cognitive, emotional and motor levels. Therefore, the particularities of this research may also be explanatory factors for the differences found.

Lastly, according to *Hypothesis 3*, participants with high levels of aggressive behavior reported significantly lower levels of PA as compared to those having *Low Aggression* levels. These results also coincide with previous research that has found a negative and significant association between aggressive behavior and PA (Hewig et al., 2004; Dufey and Fernández, 2012; Shachar et al., 2016). Thus, this negative relationship could be explained by the fact that individuals who tend to show positive emotional states usually present greater prosocial behavior (Aknin et al., 2018). In contrast, literature on aggression has concluded that individuals tend to attack others when experiencing negative emotions (Verona et al., 2002; Harmon-Jones, 2003; Dufey and Fernández, 2012; Shachar et al., 2016). In other words, “when people feel bad, they are too likely to have angry feelings, hostile thoughts and memories, and aggressive inclinations” (Berkowitz, 2001, p.335). According to this statement, results from this study have found that *High Aggression* scored significantly higher on NA than the other profiles, with these differences being moderate in size.

## Limitations and Future Research

Certain limitations of this study should be considered. First, the sampling procedure and the sample size ensure the representativeness of the Spanish community population between the ages of 8 and 12. Nevertheless, results from this study must be generalized with caution. Therefore, it would be interesting for future research to replicate this study using other age and cultural groups. Second, the design employed impedes the establishment of causal relationships between aggressive behavior and perfectionism, school refusal and affect. This limitation could be solved by using longitudinal data or with structural equation modeling. Third, this work also has the limitations of using self-report measures (Fernández-Montalvo and Echeburúa, 2006) which could be solved with a multi-source and multi-method assessment. Furthermore, it should be noted that this study does not take into account the potential relationship between perfectionism, school refusal and affect. This limitation should be addressed by future research performing a causal-explanatory model of aggression behavior considering the possible links between the predictor variables (i.e., perfectionism, school refusal and affect). Finally, it should be noted that this study is based on the Buss and Perry model which defines aggressive behavior as a set of three components: emotional, cognitive and motor. Thus, other functions such as proactive/reactive aggression have not been considered.

## Practical Implications and Conclusions

Despite the mentioned limitations, this work is a novel contribution for research on aggression for several reasons. First, it is the first study to examine profiles of aggressive behavior while jointly considering the dimensions of Anger, Hostility, Physical Aggression and Verbal Aggression. Second, no previous study has analyzed the link between aggressive behavior (according to its three components: emotional, cognitive and motor) and SOP and Positive and NA in child population. Last but not least, this is the first work to offer empirical evidence on the relationship between aggressive behavior and school refusal. Thus, in accordance with our results, almost 50% of the child population manifests high levels of Anger, Hostility and Physical and Verbal Aggression. These children also present clear perfectionist trends. They often feel sad, afraid and miserable and rarely experience positive emotions like happiness or pride. Furthermore, they tend to refuse school since attending school causes them great discomfort, because they suffer in social or school evaluation situations, or given that they have difficulties in being separated from their parents. This high aggression profile could be detected by school counselors using the AQ and identifying those students who report high scores in all dimensions evaluated by the mentioned scale.

Finally, if the correlates of aggressive behavior, perfectionism, school refusal and NA with certain problems and psychopathologies are taken into account (e.g., Jaafar et al., 2013; Thornton et al., 2013; Morris and Lomax, 2014; Schütz et al., 2014), *High Aggression* could be considered a profile having a high risk of psychological vulnerability to be treated without further delay. Therefore, Farrington et al. (2017) described the effectiveness of developmental prevention programs on aggressive behavior in children and adolescents. These programs are defined as community-based programs that may be focused on individual (providing training in social competencies, interpersonal problem solving and other cognitive or behavioral skills), family (providing counseling on child-rearing, coping with family stress or training in parenting skills) or school (improving the school climate, teaching behavior, etc.). Likewise, prevention programs on aggression should be combined with strategies designed to bolster levels of resilience in order to avoid or overcome psychological problems (i.e., perfectionism, school refusal and NA) associated with children having a *High Aggression* profile.

## Author Contributions

MV and CG have participated in conducting a literature search and writing this manuscript. CJI has reviewed this research. RS has participated in performing statistical analyses. JMG-F has designed this research.

## Conflict of Interest Statement

The authors declare that the research was conducted in the absence of any commercial or financial relationships that could be construed as a potential conflict of interest.

## References

[B1] Aknin L. B. Van de Vondervoort J. W. Hamlin J. K. (2018). Positive feelings reward and promote prosocial behavior. Curr. Opin. Psychol. 20, 55–59. 10.1016/j.copsyc.2017.08.017 28837957

[B2] Archer J. (2009). Does sexual selection explain human sex differences in aggression? Behav. Brain Sci. 32, 249–266. 10.1017/s0140525x09990951 19691899

[B4] Blair R. J. R. (2016). The neurobiology of impulsive aggression. J. Child. Adolesc. Psychopharmacol. 26, 4–9. 10.1089/cap.2015.0088 26465707 PMC4779272

[B5] Buss A. H. Perry M. (1992). The aggression questionnaire. J. Pers. Soc. Psychol. 63, 452–459. 10.1037/0022-3514.63.3.452 1403624

[B80] Castro J. Gila A. Gual P. Lahortiga F. Saura B. Toro J. (2004). Perfectionism dimensions in children and adolescents with anorexia nervosa. J. Adolesc. Health 35, 392–398. 10.1016/j.jadohealth.2003.11.094 15488433

[B6] Clark L. A. Watson D. Mineka S. (1994). Temperament, personality, and the mood and anxiety disorders. J. Abnorm. Psychol. 103, 103–116. 10.1037/0021-843X.103.1.103 8040472

[B7] Cohen J. (1988). Statistical Power Analysis for the Behavioral Sciences. Hillsdale, NJ: Erlbaum.

[B8] Donahue J. J. Gorason A. C. McClure K. S. Van Male L. M. (2014). Emotion dysregulation, negative affect, and aggression: a moderated, multiple mediator analysis. Pers. Individ. Dif. 70, 23–28. 10.1016/j.paid.2014.06.009

[B9] Dufey M. Fernández A. M. (2012). Validity and reliability of the positive affect and negative affect schedule (PANAS) in chilean college students. Rev. Iberoamericana Diagnostico Eval. Psicol. 1, 157–173.

[B10] Ebesutani C. Regan J. Smith A. Reise S. Higa-McMillan C. Chorpita B. F. (2012). The 10-item positive and negative affect schedule for children, child and parent shortened versions: application of item response theory for more efficient assessment. J. Psychopathol. Behav. Assess. 34, 191–203. 10.1007/s10862-011-9273-2

[B11] Egan S. J. Wade T. D. Shafran R. (2011). Perfectionism as a transdiagnostic process: a clinical review. Clin. Psychol. Rev. 31, 203–212. 10.1016/j.cpr.2010.04.009 20488598

[B12] Egan S. J. Wade T. D. Shafran R. (2012). The transdiagnostic process of perfectionism. Rev. Psicopatol. Psicol. Clín. 17, 279–294. 10.5944/rppc.vol.17.num.3.2012.11844

[B13] Fabiansson E. C. Denson T. F. (2016). “Anger, hostility and anger management,” in Encyclopedia of Mental Health ed. Friedman H. (Waltham, MA: Academic Press), 64–67.

[B17] Farrington D. P. Gaffney H. Lösel F. Ttofi M. M. (2017). Systematic reviews of the effectiveness of developmental prevention programs in reducing delinquency, aggression, and bullying. Aggress. Violent Behav. 33, 91–106. 10.1016/j.avb.2016.11.003

[B14] Fernández-Montalvo J. Echeburúa E. (2006). Uso y abuso de los autoinformes en la evaluación de los trastornos de personalidad. Rev. Psicopatol. Psicol. Clin. 11, 1–12. 10.5944/rppc.vol.11.num.1.2006.4014

[B15] Flett G. F. Hewitt P. L. Besser A. Su C. Vaillancourt T. Boucher D. (2016). The child-adolescent perfectionism scale: development, psychomeric properties, and associations with stress, distress and psychoatric symptoms. J. Psychoeduc. Assess. 34, 634–652. 10.1177/0734282916651381

[B16] Freeman J. Simonsen B. (2015). Examining the impact of policy and practice interventions on high school dropout and school completion rates: a systematic review of the literature. Rev. Educ. Res. 85, 205–248. 10.3102/0034654314554431

[B18] Garaigordobil M. Martínez-Valderrey V. Aliri J. (2013). Autoestima, empatía y conducta agresiva en adolescentes víctimas de bullying presencial. Eur. J. Invest. Health 3, 29–40. 10.1989/ejihpe.v3i1.21

[B19] García-Fernández J. M. Inglés C. J. Vicent M. Gonzálvez C. Gómez-Núñez M. I. Poveda-Serra P. (2016). Perfeccionismo durante la infancia y la adolescencia. Análisis bibliométrico y temático (2004–2014). Rev. Iberoamericana Psicol. Salud 7, 79–88. 10.1016/j.rips.2016.02.001

[B20] García-Fernández J. M. Vicent M. Inglés C. J. Gonzálvez C. Sanmartín R. (2017). Relación entre el perfeccionismo socialmente prescrito y la conducta agresiva durante la infancia tardía. Eur. J. Educ. Psychol. 10, 15–22. 10.1016/j.ejeps.2016.10.003

[B22] Gonzálvez C. Inglés C. J. Kearney C. A. Vicent M. Sanmartín R. García-Fernández J. M. (2016). School refusal assessment scale-revised: factorial invariance and latent means differences across gender and age in Spanish children. Front. Psychol. 7:2011. 10.1177/0734282917712173 28082938 PMC5183572

[B23] Grant K. E. Compas B. E. Thurm A. E. McMahon S. D. Gipson P. Y. (2004). Stressors and child and adolescent psychopathology: measurement issues and prospective effects. J. Clin. Child Adolesc. Psychol. 33, 412–425. 10.1207/s15374424jccp3302_23 15136206

[B24] Hambleton R. K. Lee M. K. (2015). “Methods for translating and adapting tests to increase cross-language validity”, in The Oxford Handbook of Child Psychological Assessment, eds Saklofske D. H. Reynolds C. R. Schwean V. L. (New York, NY: Oxford University Press), 172–181.

[B25] Hampson S. E. Tildesley E. Andrews J. A. Luyckx K. Mroczek D. K. (2010). The relation of change in hostility and sociability during childhood to substance use in mid adolescence. J. Res. Pers. 44, 103–114. 10.1016/j.jrp.2009.12.006 20401178 PMC2854561

[B26] Harachi T. W. Fleming C. B. White H. R. Ensminger M. E. Abbott R. D. Catalano R. F. (2006). Aggressive behavior among girls and boys during middle childhood: predictors and sequelae of trajectory group membership. Aggress. Behav. 32, 279–293. 10.1002/ab.20125

[B27] Harmon-Jones E. (2003). Anger and the behavioural approach system. Pers. Individ. Dif. 35, 995–1005. 10.1016/S0191-8869(02)00313-6

[B28] Hay D. F. (2017). The early development of human aggression. Child Dev. Perspect. 11, 102–106. 10.1111/cdep.12220

[B29] Hewig J. Hagemann D. Seifert J. Naumann E. Bartussek D. (2004). On the selective relation of frontal cortical asymmetry and anger-out, versus anger-control. J. Pers. Soc. Psychol. 87, 926–939. 10.1037/0022-3514.87.6.926 15598115

[B30] Higa C. K. Daleiden E. L. Chorpita B. F. (2002). Psychometric properties and clinical utility of the school refusal assessment scale in a multi-ethnic sample. J. Psychopathol. Behav. Assess. 24, 247–258. 10.1023/A:1020727016113

[B31] Inglés C. J. Gonzálvez-Maciá C. García-Fernández J. M. Vicent M. Martínez-Montegudo M. C. (2015). Current status of research on school refusal. Eur. J. Educ. Psychol. 8, 37–52. 10.1016/j.ejeps.2015.10.005

[B32] Ingul J. M. Nordahl H. M. (2013). Anxiety as a risk factor for school absenteeism: what differentiates anxious school attenders from non-attenders? Ann. Gen. Psychiatry 12:25. 10.1186/1744-859x-12-25 23886245 PMC3726429

[B33] Jaafar N. R. N. Irayani M. D. T. Salwina W. I. W. Nazri A. R. F. Kamal N. A. Prakash R. J.. (2013). Externalizing and internalizing syndromes in relation to school truancy among adolescents in high-risk urban schools. Asia Pac. Psychiatry 5, 27–34. 10.1111/appy.12072 23857834

[B34] Kearney C. A. (2002). Identifying the function of school refusal behavior: a revision of the school refusal assessment scale. J. Psychopathol. Behav. Assess. 24, 235–245. 10.1023/A:1020774932043

[B35] Kearney C. A. (2016). Managing School Absenteeism at Multiple Tiers. An Evidence-Based and Practical Guide for Professionals. New York, NY: Oxford University Press.

[B36] Kearney C. A. Albano A. (2004). The functional profiles of school refusal behavior: diagnostic aspects. Behav. Modif. 28, 147–161. 10.1177/0145445503259263 14710711

[B37] Kearney C. A. Bensaheb A. (2006). School absenteeism and school refusal behavior: a review and suggestions for school-based health professionals. J. Sch. Health 76, 3–7. 10.1111/j.1746-1561.2006.00060.x 16457678

[B40] Kearney C. A. Chapman G. Cook L. C. (2005). School refusal behavior in young children. Int. J. Behav. Consult. Ther. 1, 216–222. 10.1037/h0100746

[B38] Kearney C. A. Diliberto R. (2014). “School refusal behaviour”, in The Wiley Handbook of Cognitive Behavioral Therapy, eds Hofmann S. G. Rief W. (New York, NY: Wiley), 875–892.

[B39] Kearney C. A. Silverman W. K. (1993). Measuring the function of school refusal behavior: the school refusal assessment scale. J. Clin. Child Psychol. 22, 85–96. 10.1207/s15374424jccp2201_9

[B41] Kerr M. A. Schneider B. H. (2008). Anger expression in children and adolescents: a review of the empirical literature. Clin. Psychol. Rev. 28, 559–577. 10.1016/j.cpr.2007.08.001 17884263

[B42] Leary M. R. Twenge J. M. Quinlivan E. (2006). Interpersonal rejection as a determinant of anger and aggression. Pers. Soc. Psychol. Rev. 10, 111–132. 10.1207/s15327957pspr1002_2 16768650

[B43] Liu Y. Wang Z. Zhou C. Li T. (2014). Affect and self-esteem as mediators between trait resilience and psychological adjustment. Pers. Individ. Dif. 66, 92–97. 10.1016/j.paid.2014.03.023

[B44] Lubke G. H. Ouwens K. G. de Moor M. Trull T. Boomsma D. I. (2015). Population heterogeneity of trait anger and differential associations of trait anger facets with borderline personality features, neuroticism, depression, attention deficit hyperactivity disorder (ADHD), and alcohol problems. Psychiatry Res. 230, 553–560. 10.1016/j.psychres.2015.10.003 26454404 PMC4655156

[B45] Maynard B. R. Salas-Wright C. P. Vaughn M. G. Peters K. E. (2012). Who are truant youth? Examining distinctive profiles of truant youth using latent profile analysis. J. Youth Adolesc. 41, 1671–1684. 10.1007/s10964-012-9788-1 22766683 PMC3653415

[B46] McKay M. T. Perry J. L. Harvey S. A. (2016). The factorial validity and reliability of three versions of the aggression questionnaire using confirmatory factor analysis and exploratory structural equation modeling. Pers. Individ. Dif. 90, 12–15. 10.1016/j.paid.2015.10.028

[B47] Modecki K. L. Michin J. Harbaugh A. G. Guerra N. G. Runions K. C. (2014). Bullying prevalence across contexts: a meta-analysis measuring cyber and traditional bullying. J. Adolesc. Health 55, 602–611. 10.1016/j.jadohealth.2014.06.007 25168105

[B48] Morris L. Lomax C. (2014). Review: assessment, development, and treatment of childhood perfectionism: a systematic review. Child Adolesc. Ment. Health 19, 225–234. 10.1111/camh.12067 32878354

[B49] Nelson R. J. Trainor B. C. (2007). Neural mechanisms of aggression. Nat. Rev. Neurosci. 8, 536–546. 10.1038/nrn2174 17585306

[B50] Nyland K. L. Asparouhov T. Muthén B. O. (2007). Deciding on the number of classes in latent class analysis and growth mixture modelling: a monte carlo simulation study. Struct. Equ. Modeling 14, 535–569. 10.1080/10705510701575396

[B51] Pellegrini A. D. (2007). “Is aggression adaptive? Yes: some kinds are and in some ways”, in Aggression and Adaptation: the Bright Side to Bad Behavior, eds Hawley P. H. Little T. D. Rodkin P. C. (Mahwah, NJ: Lawrence Erlbaum Associates), 85–105.

[B52] Petersen I. T. Bates J. E. Dodge K. A. Lansford J. E. Pettit G. S. (2015). Describing and predicting developmental profiles of externalizing problems from childhood to adulthood. Dev. Psychopathol. 27, 791–818. 10.1017/s0954579414000789 25166430 PMC4344932

[B53] Piquero A. R. Carriaga M. L. Diamond B. Kazemian L. Farrington D. P. (2012). Stability in aggression revisited. Aggress. Violent Behav. 17, 365–372. 10.1016/j.avb.2012.04.001

[B55] Santisteban C. Alvarado J. M. (2009). The aggression questionnaire for Spanish preadolescents and adolescents: AQ-PA. Span. J. Psycho. 12, 320–326. 10.1017/s1138741600001712 19476243

[B56] Schreiber J. B. (2017). Latent class analysis: an example for reporting results. Res. Social Adm. Pharm. 13, 1196–1201. 10.1016/j.sapharm.2016.11.011 27955976

[B58] Schütz E. Garcia D. Archer T. (2014). Affective state, stress and type a-personality as a function of gender and affective profiles. Int. J. Res. Stud. Psychol. 3, 51–64. 10.5861/ijrsp.2013.450

[B59] Schütz E. Sailer U. Nima A. A. Rosenberg P. Arntén A. C. A. Archer T.. (2013). The affective profiles in the USA: happiness, depression, life satisfaction and happiness-increasing strategies. PeerJ 1:e156. 10.7717/peerj.156 24058884 PMC3775633

[B60] Serafini G. Pompili M. Innamorati M. Rihmer Z. Sher L. Girardi P. (2012). Increase the suicide risk in psychosis? A critical review. Curr. Pharm. Des. 18, 5165–5187. 10.2174/138161212802884663 22716157

[B61] Shachar K. Ronen-Rosenbaum T. Rosenbaum M. Orkibi H. Hamama L. (2016). Reducing child aggression through sports intervention: the role of self-control skills and emotions. Child Youth Serv. Rev. 71, 241–249. 10.1016/j.childyouth.2016.11.012

[B62] Smeets K. C. Oostermeijer S. Lappenschaar M. Cohn M. van der Meer J. M. M. Popma A.. (2017). Are proactive and reactive aggression meaningful distinctions in adolescents? A variable- and person-based approach. J. Abnorm. Child Psychol. 45, 1–14. 10.1007/s10802-016-0149-5 27113216 PMC5219021

[B63] Stoeber J. Noland A. B. Mawenu T. W. N. Henderson T. M. Kent D. N. P. (2017). Perfectionism, social disconnection and interpersonal hostility: not all perfectionists don’t play nicely with others. Pers. Individ. Dif. 119, 112–117. 10.1016/j.paid.2017.07.008

[B64] Thornton M. Darmody M. McCoy S. (2013). Persistent absenteeism among Irish primary school pupils. Educ. Rev. 65, 488–501. 10.1080/00131911.2013.768599

[B65] Verona E. Patrick C. J. Lang A. R. (2002). A direct assessment of the role of state and trait negative emotion in aggressive behavior. J. Abnorm. Psychol. 111, 249–258. 10.1037/0021-843x.111.2.249 12003447

[B66] Vicent M. Inglés C. J. Sanmartín R. Gonzálvez C. García-Fernández J. M. (2017). Perfectionism and aggression: identifying risk profiles in children. Pers. Individ. Dif. 112, 106–112. 10.1016/j.paid.2017.02.061

[B67] Wallinius M. Delfin C. Billstedt E. Nilsson T. Anckarsäter H. Hofvander B. (2016). Offenders in emerging adulthood: school maladjustment, childhood adversities and prediction of aggressive antisocial behaviors. Law Hum. Behav. 40, 551–563. 10.1037/lhb0000202 27243360

[B68] Watson D. Clark L. A. Tellegen A. (1988). Development and validation of brief measures of positive and negative affect: the PANAS scales. J. Pers. Soc. Psychol. 54, 1063–1070. 10.1037/0022-3514.54.6.1063 3397865

[B69] Yahaya A. Ramli J. Hashim S. Ibrahim M. A. Kadir H. B. H. Boon Y. (2010). The effects of various modes of absenteeism problem in school on the academic performance of students in secondary schools. Eur. J. Soc. Secur. 12, 624–639.

